# *SLC12A7* alters adrenocortical carcinoma cell adhesion properties to promote an aggressive invasive behavior

**DOI:** 10.1186/s12964-018-0243-0

**Published:** 2018-06-08

**Authors:** Taylor C. Brown, Timothy D. Murtha, Jill C. Rubinstein, Reju Korah, Tobias Carling

**Affiliations:** 1 Department of Surgery, Yale University School of Medicine, 333 Cedar Street, TMP FMB130A, P.O. Box 208062, New Haven, CT 06520 USA; 2 Yale Endocrine Neoplasia Laboratory, Yale University School of Medicine, 333 Cedar Street, TMP FMB130A, P.O. Box 208062, New Haven, CT 06520 USA

**Keywords:** Adrenocortical carcinoma, SLA12A7, Ezrin, Filopodia

## Abstract

**Background:**

Altered expression of Solute Carrier Family 12 Member 7 (SLC12A7) is implicated to promote malignant behavior in multiple cancer types through an incompletely understood mechanism. Recent studies have shown recurrent gene amplifications and overexpression of SLC12A7 in adrenocortical carcinoma (ACC). The potential mechanistic effect(s) of SLC12A7 amplifications in portending an aggressive behavior in ACC has not been previously studied and is investigated here using two established ACC cell lines, SW-13 and NCI-H295R.

**Methods:**

SW-13 cells, which express negligible amounts of SLC12A7, were enforced to express SLC12A7 constitutively, while RNAi gene silencing was performed in NCI-H295R cells, which have robust endogenous expression of SLC12A7. In vitro studies tested the outcomes of experimental alterations in SLC12A7 expression on malignant characteristics, including cell viability, growth, colony formation potential, motility, invasive capacity, adhesion and detachment kinetics, and cell membrane organization. Further, potential alterations in transcription regulation downstream to induced SLC12A7 overexpression was explored using targeted transcription factor expression arrays.

**Results:**

Enforced SLC12A7 overexpression in SW-13 cells robustly promoted motility and invasive characteristics (*p* < 0.05) without significantly altering cell viability, growth, or colony formation potential. SLC12A7 overexpression also significantly increased rates of cellular attachment and detachment turnover (*p* < 0.05), potentially propelled by increased filopodia formation and/or Ezrin interaction. In contrast, RNAi gene silencing of *SLC12A7* stymied cell attachment strength as well as migration and invasion capacity in NCI-H295R cells. Transcription factor expression analysis identified multiple signally pathways potentially affected by SLC12A7 overexpression, including osmotic stress, bone morphogenetic protein, and Hippo signaling pathways.

**Conclusions:**

Amplification of SLC12A7 observed in ACCs is shown here, in vitro, to exacerbate the malignant behavior of ACC cells by promoting invasive capacities*,* possibly mediated by alterations in multiple signaling pathways, including the osmotic stress pathway.

## Plain english summary

Adrenal cancer is a very deadly cancer and current treatment options are limited, especially for patients with cancers that have spread throughout the body. Previous research discovered increased expression of a gene called *Solute Carrier Family 12 Member 7* (*SLC12A7*). However, no studies have been performed to test whether SLC12A7 is involved in promoting adrenal cancers, and if so, the mechanisms behind its potential effect. Using two well-studied adrenal cancer cell lines, one with very low expression of SLC12A7 (SW-13 cells), and one with very high expression of SLC12A7 (NCI-H295R cells), we addressed these questions in this study.

Results of experiments in this study clearly show that when SLC12A7 expression was increased, the cells became more aggressive. Similarly, suppressing SLC12A7 function resulted in the opposite response. Moreover, experiments showed that SLC12A7 promotes the malignant behavior of adrenal cancer cells by changing their cell membrane attachment kinetics and consequently, accelerates their invasive behavior. Additional experiments also suggest a role for osmotic stress as a driving force for these changes.

In conclusion, this study demonstrates that SLC12A7 plays an important role in adrenal cancer by promoting tumor invasion, and as such, could represent a marker of tumor aggressiveness to serve as a potential therapeutic target.

## Background

Adrenocortical carcinoma (ACC) is a rare and an aggressive cancer. Its estimated incidence ranges from 0.7 to 2.0 cases per million people per year [1]. Tumor occurrence has a bimodal age distribution, with the first peak observed in childhood and a second, higher peak observed during the 5th and 6th decades of life [2, 3]. Patients usually present with symptoms of abdominal pain and/or excessive hormone production, while some are incidentally diagnosed by cross-sectional imaging [3]. For patients with European Network for the Study of Adrenal Tumors (ENSAT) stage I-III tumors surgery is the primary treatment modality, with *en bloc*, R0 resection providing the only chance for cure. In addition to surgery, external beam radiation, Mitotane, chemotherapy, and targeted therapies are used as either primary, or as adjuvant therapy [4].

Three molecular pathways are frequently found disrupted in ACC and play a significant role in tumor origin and/or progression. Activation of canonical Wnt/beta-catenin signaling occurs frequently in ACC via mutations of the proto-oncogene *CTNNB1* (*β-Catenin*), which in turn stimulates target gene transcription and promotes tumor formation [5]. Somatic mutations in the tumor suppressor gene *TP53* is found in approximately 20–35% of cases and are associated with more aggressive tumors. Furthermore, Li Fraumeni Syndrome, which is caused by germline *TP53* mutations, is often associated with childhood ACCs [1, 3]. Overexpression of insulin growth factor II (IGF-II) via alteration of gene copy number and/or gene imprinting is one of the most frequently observed molecular events associated with ACC [3, 5].

Gene copy number variations (CNVs) occur frequently in ACC and promote the malignant development of these tumors [6–10]. Two studies utilizing whole-exome sequencing (WES) methods identified the 5p13.33 chromosome location to be the most recurrently amplified region in the ACC genome [11, 12]. *Solute Carrier Family 12 Member 7* (*SLC12A7*), a recently characterized proto-oncogene, is found at this location. It has been shown that *SLC12A7* gene copy gains in ACC promote mRNA and protein overexpression and is associated with non-functional tumors [13].

SLC12A7 (KCl cotransporter 4; KCC4), a member of the *SLC12* gene family, is a 1083 amino acid long, trans-membrane protein that regulates cell volume via potassium and chloride transport [14, 15]. However, it has also been demonstrated that amplified expression of SLC12A7 promotes the malignant behavior of several different cancer types. SLC12A7 is overexpressed in gynecological and breast cancers and overexpression of SLC12A7 and other SLC12 gene family members has been shown to be associated with local tumor invasion, lymph node metastases, and poor clinical outcomes. Furthermore, SCL12A7 has been shown to promote in vitro tumor cell invasion [16–19], potentially mediated through interactions with Ezrin (EZR), a membrane cytoskeleton/extra-cellular matrix linker [19]. Based on the previous findings by our group and others, we sought to determine the phenotypic effects of SLC12A7 overexpression upon ACC malignant behavior.

## Methods

### Cell culture, vector transfection, RNAi gene silencing, gene expression analysis, and Western blot detection

ACC cell culture and vector transfection were performed as previously described [20]. Briefly, the human ACC cell lines SW-13 and NCI-H295R (authenticated and supplied by American Type Cell Collection) were maintained under sterile conditions in a humidified incubator at 37.0 C with 5% CO_2_. SW-13 cells were grown in Dulbecco’s Modified Eagle Medium (DMEM) supplemented with 10% certified fetal bovine serum (FBS) and 10,000 U/mL penicillin/streptomycin; designated as complete medium (CM). NCI-H295R cells were grown in DMEM/F12 supplemented with 5% NuSerum, 10,000 U/mL penicillin/streptomycin, 5 μg/ml of insulin, 5 μγ/ml of transferrin, and 5 ng/ml of selenium (all reagents from Applied Biosystems); designated complete medium as well (CM). In general, cell strains underwent no more than 10 passages before experiments were performed.

Myc-DDK tagged pCMV6-Entry and pCMV6-Entry/SLC12A7-ORF plasmid expression vectors (Origene) were transfected into SW-13 cells using Lipofectamine 3000 (ThermoFisher) according to the manufacturer’s recommendations in 6-well plates with cells grown to 70–80% confluence. Stable clones of pCMV6-Entry and pCMV6-Entry/SLC12A7 vectors were selected in CM containing 800 μg/ml G-418 (Life Technologies). Multiple clones were then pooled into populations to avoid clonal variability. Selected SW-13 cell populations were designated SW-13/V (pCMV6 vector-transfected) and SW-13/S (pCMV6/SLC12A7-transfected) and were utilized to determine the effects of constitutive overexpression of SLC12A7 on the malignant behavior of SW-13 cells. Parental, un-transfected SW-13 cells were used as an additional reference control.

RNAi gene silencing of NCI-H295R cells were carried out with 3 unique 27-mer siRNA duplexes (designated siA, siB, and siC) targeting *SLC12A7* (Human) using the standard protocol as previously described [21]. Universal scrambled negative control siRNA was used as non-specific control (all from Origene). Lipofectamine 3000-mediated transfection was carried out in Opti-MEM medium according to the manufacturer’s recommendations (ThermoFisher) in 6-well plates with starting densities of 100,000 cells/well. Transfection medium was replaced with CM after 6 h of transfection. Cells were lysed for RNA extraction and gene expression analysis at 24 h post-transfection.

De novo and altered expression levels of *SLC12A7*, *CEBPG*, *ID1*, *NFAT5*, and *SMAD5* mRNA were determined by gene expression analysis using a TaqMan assay (Applied Biosystems). Briefly, RNA was isolated from cells using the RNeasy Plus Mini Kit (Qiagen). Quantity and quality of isolated RNA was assessed by spectrophotometry (NanoDrop Technologies). Two hundred ng of RNA was used for cDNA synthesis using the iScript cDNA synthesis kit (Bio-Rad). Real-time quantitative PCR was performed on a CFX96 Real-Time System thermal cycler (Bio-Rad) using TaqMan PCR master mix (Applied Biosystems) with primers and probes (Applied Biosystems) specific to target genes. Relative expression was quantified using expression levels of housekeeping gene *ribosomal protein large P0* (*RPLP0*) as a reference control (Hs99999902_m1; Applied Biosystems). Relative gene expressions were calculated using the Livak method [22]. Gene expression analysis was performed in triplicate.

Enforced overexpression of SLC12A7 protein in SW-13 cells was confirmed via Western blot technique using anti-SLC12A7 rabbit polyclonal antibody (Novus), anti-rabbit-HRP goat antibody (Santa Cruz Biotech), mini-PROTEAN TGX gel (BioRad), PVDF blotting membrane (BioRad), and enhanced chemiluminescence detection reagents (ThermoFisher Scientific) according to the manufacturer’s protocols. Equivalent protein loading was ensured via Western blot detection of beta-actin expression using anti-β−actin mouse monoclonal antibody followed by anti-mouse-HRP goat secondary antibody (Santa Cruz Biotech). Western blot analysis was performed in duplicate.

### Cell growth and clonogenic assays

Cell growth and clonogenic analysis was performed as previously described [20]. Briefly, for growth assays, 50,000 cells per well of SW-13, SW-13/V, and SW-13/S cells were plated in 6-well plates and grown in CM at 37 °C. Total cell numbers and viability were assessed by staining cells with 0.4% Trypan Blue (Life Technology) and counting cells using a hemocytometer (Hausser Scientific) each day for 6 days. For clonogenic assays, 5000 cells per well of SW-13, SW-13/V, and SW-13/S were plated in 6-well plates and allowed to grow for 5 days in CM at 37 °C. On day 6, cells were washed with phosphate-buffered saline (PBS), fixed with 3.7% formaldehyde for 10 min, stained with 0.05% crystal violet for 30 min, washed in deionized water, and tabulated under microscopy. Independent colonies with 8 or more cells were counted and averaged from 6 wells. Cell and colony growth analysis was performed in 2 independent experiments with 6 wells for each cell type analyzed.

### Migration and invasion assay

Migration and invasion analyses were performed as previously described [20]. For migration, 100,000 cells of SW-13, SW-13/V, SW-13/S, and NCI-H295R were allowed to migrate through 8 μM pores in modified Boyden chambers (BD Biosciences) from upper chambers containing serum-free medium to lower chambers containing CM at 37 °C. After designated time points, cells that migrated to the lower side of the membrane towards a higher FBS concentration were fixed in 3.7% formaldehyde for 10 min, stained with 0.05% crystal violet for 30 min, washed using deionized water, and tabulated under microscopy. For invasion, 100,000 cells of SW-13, SW-13/V, SW-13/S, and NCI-H295R were allowed to invade in modified Boyden chambers from upper chambers containing serum-free medium through a layer of reconstituted Matrigel (BD Biosciences), which functions as a surrogate for the extracellular matrix, to lower chambers containing CM at 37 °C. After 24 h, the Matrigel was removed and cells that invaded through the Matrigel and migrated to the lower side of the membrane were fixed, stained, washed, and tabulated as above. Migration and invasion assays were performed in 2 independent experiments using 3 wells per each cell type analyzed.

### Adhesion and detachment assay

For cell adhesion assays, 10,000 cells per well of SW-13, SW-13/V, SW-13/S or 250,000 cells per well of NCI-H295R were seeded in 24-well plates. The cell numbers were titrated to avoid overcrowding by trial experiments. Cells were incubated in CM at 37 °C for designated time points to allow for cell adherence to well surfaces. After each time point, cells were washed twice with PBS to remove non-adhered cells. The remaining, firmly attached cells were then fixed with 3.7% formaldehyde for 10 min, stained with 0.05% crystal violet for 30 min, washed using deionized water, and tabulated under microscopy. For cell detachment assays, 100,000 cells per well of SW-13, SW-13/V, and SW-13/S cells were seeded in 24-well plates. Cells were incubated in CM at 37 °C overnight. The following day cells were detached with a cell dissociation solution containing ethylenediaminetetraacetic acid tetrasodium salt dehydrate (EDTA; Sigma Aldrich) at multiple time points up to 15 min. The remaining attached cells were then fixed, stained, washed, and tabulated as above. Adhesion and detachment assays for both cells lines were performed in 2 independent experiments using 3–4 wells per each cell type/experimental condition analyzed.

### Analysis of cell membrane organization by light and immunofluorescence microscopy

The effect of SLC12A7 overexpression on SW-13 cell extension repertoire was performed as previously described [21]. Briefly, cells were grown on glass coverslips in CM overnight, fixed with 3.7% formaldehyde, stained with 0.05% crystal violet, washed using deionized water, and photographed under 400X magnification. Cell extensions on isolated, non-overlapping cells from 20 randomly selected photomicrographs for each cell type (SW-13, SW-13/V, and SW-13/S) were classified as three major types (lamellipodia, lobopodia, and filopodia) of cell surface extensions, averaged, and tabulated. For immunofluorescence analysis, cells were grown on sterile glass cover slips in CM and after 24 h cells were fixed in cold acetone-methanol (1:1) for 10 min. Immunostaining was then performed for SLC12A7 and EZR proteins using mouse anti-SLC12A7 (Santa Cruz Biotech) and rabbit anti-EZRIN (Abcam) primary antibodies and anti-mouse FITC or anti-rabbit TR secondary antibodies (Santa Cruz Biotech), followed by UltraCruz mounting agent containing 4′,6-diamidino-2-phenylindole – DAPI (Santa Cruz Biotech). Dilutions and incubations were carried out per the manufacturer’s recommendations. A Zeiss AX10 confocal microscope with AxioVision 4.8 program was used for immunofluorescence analysis and photomicrographs were taken at a total magnification of 1000X. Analysis of cell membrane organization by light and immunofluorescence was performed in duplicate.

### Transcription factor expression array analysis

To assess possible altered transcription factor repertoire downstream to SLC12A7 enforced overexpression, transcription factor gene expression analysis was performed using the RT^2^ Profiler PCR Array Human Transcription Factors (PAHS-075Z; Qiagen) per the manufacturer’s instructions. Briefly, 500 ng of total RNA from SW-13, SW-13/V, and SW-13/S cells were purified using the DNA Elimination Mix, subjected to cDNA synthesis using the RT^2^ First Strand Kit, and then amplified with real-time quantitative PCR analysis using a CFX96 Real-Time System thermal cycler (Bio-Rad) using RT^2^ SYBR Green qPCR Mastermix (Qiagen). Using parental SW-13 cells as a reference control, relative expression levels of 84 transcription factors were analyzed with SABioscience PCR Array Data Analysis web-based software (Qiagen). Transcription factor expression array analysis was performed in duplicate. To confirm the significance of altered transcription factor signaling identified in SW-13/S cells, candidate transcription factor expression was measured using quantitative PCR techniques (refer above) in NCI-H295R cells treated for *SLC12A7* siRNA knock down.

### Statistical analysis

Continuous variables were analyzed using a two-tailed *t* test for normally distributed variables, or the Mann-Whitney *U* test for non-normally distributed variables. For variables with greater than two dependent values, a one-way analysis of variance (ANOVA) or Kruskal-Wallis tests were utilized for normally or non-normally distributed populations, respectively, with multiple comparisons made with a Tukey’s test. To assess the influence of two or more independent variables on one continuous dependent variable, a two-way ANOVA was utilized and multiple comparisons were made with a Tukey’s test. A *p*-value ≤ 0.05 was considered significant. Statistical analyses were performed using GraphPad Prism 7 (GraphPad Software).

## Results

### Expression of SLC12A7 in ACC cells

**Fig. 1:**
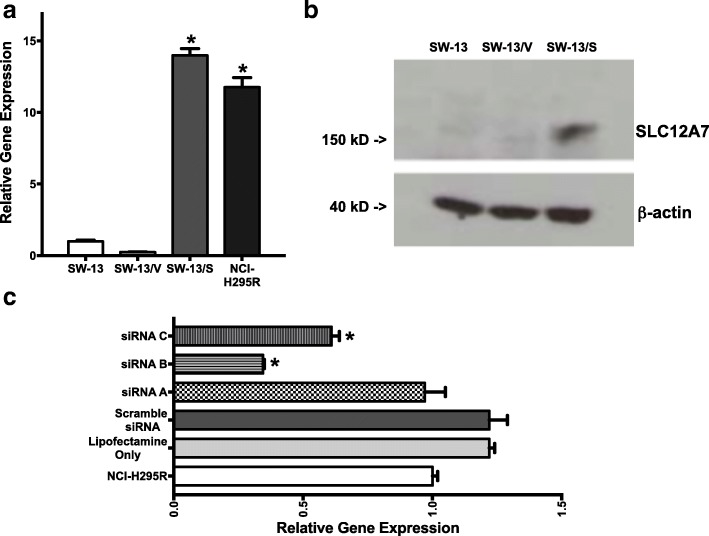
Endogenous and ectopic expression of *SLC12A7* in ACC cells. **a** Relative messenger RNA (mRNA) expression levels in adrenocortical carcinoma cells lines SW-13, SW-13/V, SW-13/S, and NCI-H295R by real-time quantitative polymerase chain reaction were determined. Expression levels were significantly higher in SW-13/S and NCI-H295R cells compared to SW-13 and SW-13/V cells (*, *p* < 0.05, one-way ANOVA). Gene expression analysis was performed in triplicate. *Horizontal bar*, mean; *Error bars*, standard error of mean. **b** Western immunoblot of SLC12A7 in SW-13, SW-13/V, and SW-13/S cells. Protein expression was higher in SW-13/S cells compared to SW-13 and SW-13/V cells. β-actin served as a loading control. Western blot analysis was performed in duplicate. **c** Relative mRNA expression levels were determined in NCI-H295 cells undergoing RNAi gene silencing by three siRNAs (siRNA-A, siRNA-B, and siRNA-C) targeting *SLC12A7* and scrambled siRNA. Significantly lower expression levels were noted in siRNAs B and C transfected cells compared to parent cells (*, *p* < 0.05, one-way ANOVA). *Horizontal bar*, mean; *Error bars*, standard error of mean. Gene expression analysis was performed in triplicate

Of the two cell lines tested in this study, NCI-H295R cells are known to contain 5p amplifications involving the *SLC12A7* locus [8], but mRNA expression patterns have not been previously tested in either cell line used in this study. Using quantitative PCR techniques, *SLC12A7* mRNA expression levels were determined. Compared to NCI-H295R cells, SW-13 cells expressed negligible amounts of *SLC12A7* mRNA transcripts (*p* < 0.05, Fig. 1a). As such, to assess the possible effects of altered expression of SLC12A7 observed in ACC tumors, SW-13 cells were stably transfected with *SLC12A7* open reading frame under a constitutively active CMV promoter (pCMV6-Entry/SLC12A7) and selected for Neomycin resistance (designated SW-13/S). Cells transfected with vector alone (pCMV6, designated SW-13/V) and parental SW-13 cells were used as controls. Expression analysis demonstrated a robust increase in mRNA *SLC12A7* transcript levels in SW-13/S cells compared to controls (SW-13/V and SW-13 cells; *p* < 0.05, Fig. 1a) and nearly equivalent with *SLC12A7* levels in NCI-H295R cells. Similarly, Western blot analysis demonstrated that SLC12A7 protein is overexpressed in SW-13/S cells compared to SW-13/V and SW-13 cells (Fig. 1b). RNAi gene silencing in NCI-H295R cells known to a have gene amplification of *SLC12A7* resulted in the suppression of *SLC12A7* mRNA levels up to 60% (siB), compared to the control siRNA treatments, including the scrambled siRNA (*p* < 0.05, Fig. 1c).

### Overexpression of SLC12A7 does not affect cell growth or colony formation

**Fig. 2:**
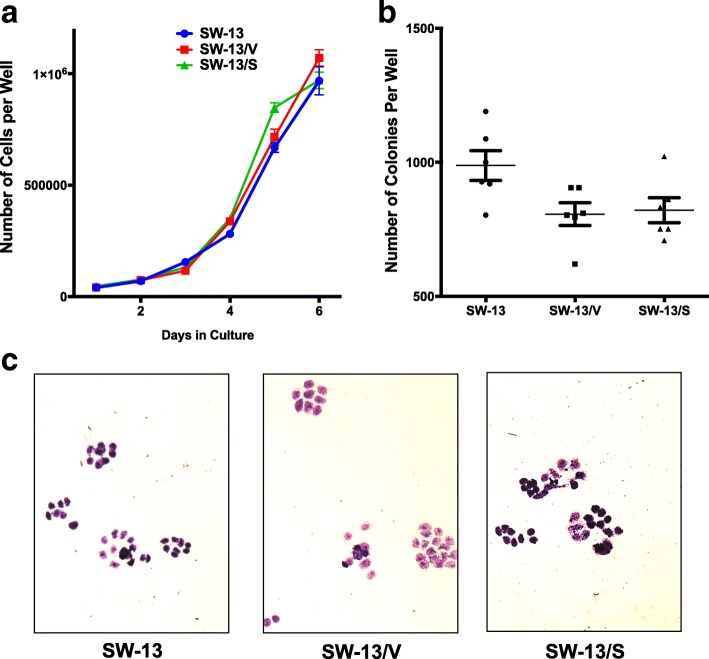
SW-13 Growth and clonogenic potential analysis. **a** SW-13, SW-13/V, and SW-13/S cells were plated with 50,000 cells per well and grown in complete medium (CM) for 6 days (with medium changed on day 3) and counted for viable and non-viable cells each day using 0.4% Trypan Blue. SLC12A7 overexpression had no significant effect on cell growth (two-way ANOVA) compared to their controls. *Horizontal bar*, mean; *Error bars*, standard error of mean. Cell growth analysis was performed in 2 independent experiments with 6 wells for each cell type analyzed. **b** SW-13, SW-13/V, and SW-13/S cells were plated at clonogenic density (5000 cells/well) and grown in CM for 6 days. Colonies with 8 or more cells were counted. SLC12A7 overexpression had no significant effect on colony growth (one-way ANOVA) compared to their controls. *Horizontal bar*, mean; *Error bars*, standard error of mean. Colony growth analysis was performed in 2 independent experiments with 6 wells for each cell type analyzed. **c** Representative photomicrographs of SW-13, SW-13/V, and SW-13/S cells after 6 days of colony growth

To assess whether constitutive overexpression of SLC12A7 promotes ACC cell growth, cell proliferation and viability were assessed during a 6-day period in SW-13, SW-13/V, and SW-13/S cells. Enforced overexpression of SLC12A7 did not significantly impact overall cell growth or viability (viability data not shown) (Fig. 2a). Clonogenic survival and growth are hallmarks of malignant tumors [20] and we tested whether overexpression of SLC12A7 impacted ACC clonogenic growth potential. As shown in Fig. 2b and c, SLC12A7 overexpression also did not significantly alter clonogenic potential in SW-13/S cells compared to SW-13 and SW-13/V cells. Growth and clonogenic survival were not tested in NCI-H295R cells due to the slow growth rate and the transient effect of RNAi gene silencing.

### SLC12A7 promotes cell migration and invasion

**Fig. 3:**
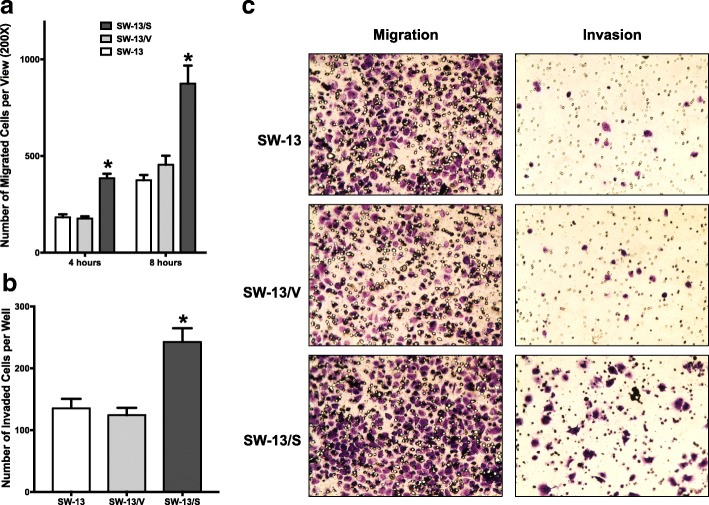
SW-13 Migration and invasion analysis. **a** For 4 and 8 h, 100,000 cells per well of SW-13, SW-13/V, and SW-13/S were allowed to migrate through a modified Boyden chamber. Increased migration rates at both time points were in observed in SW-13/S cells (*, *p* < 0.05, two-way ANOVA). *Horizontal bar*, mean; *Error bars*, standard error of mean. Migration assays were performed in two independent experiments using 3 wells per each cell type analyzed. **b** One hundred thousand cells of SW-13, SW-13/V, and SW-13/S were allowed to invade through a Matrigel layer in a modified Boyden chamber. Increased invasion activity was noted in SW-13/S cells (*, *p* < 0.05, one-way ANOVA). *Horizontal bar*, mean; *Error bars*, standard error of mean. Invasion assays were performed in two independent experiments using 3 wells per each cell type analyzed. **c** Representative photomicrographs of migrating and invading SW-13/S cells with controls are shown

**Fig. 4:**
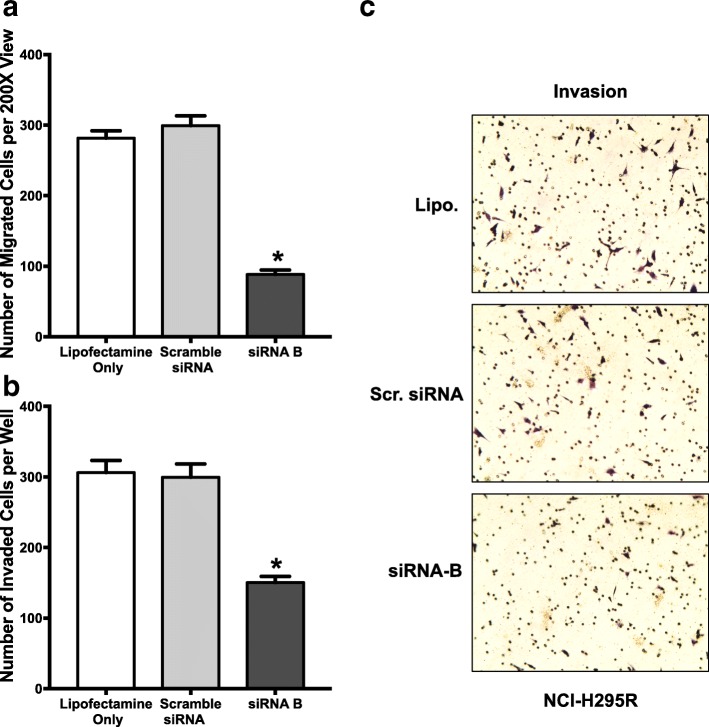
**a** One hundred thousand cells of NCI-H295R cells were allowed to migrate through a Boyden chamber. Decreased migration activity was noted in NCI-H295R cells undergoing *SLC12A7* knock down with siRNA B duplex (*, *p* < 0.05, one-way ANOVA). *Horizontal bar*, mean; *Error bars*, standard error of mean. Migration assays were performed in two independent experiments using 3 wells per each experimental condition analyzed. **b** One hundred thousand cells of NCI-H295R cells were allowed to invade through a Matrigel layer in a modified Boyden chamber. Decreased invasion activity was noted in NCI-H295R cells undergoing *SLC12A7* knock down with siRNA B duplex (*, *p* < 0.05, one-way ANOVA). *Horizontal bar*, mean; *Error bars*, standard error of mean. Invasion assays were performed in two independent experiments using 3 wells per each experimental condition analyzed. **c** Representative photomicrographs of NCI-H295R invasion undergoing siRNA knockdown are shown right

Previous studies have demonstrated that SLC12A7 promotes tumor cell metastasis, in part, by stimulating cell migration and invasion [18, 19]. Using a modified Boyden chamber assay, we tested whether alterations in SLC12A7 expression levels affected cell migration. Overexpression of SLC12A7 in SW-13 cells was found to robustly promote cellular migration at multiple time points, with an approximate 50% increase in migration rate observed in SW-13/S cells at four and 8 h (*p* < 0.05; Fig. 3a & left panel, Fig. 3c). SLC12A7 overexpression also promoted cell invasion through Matrigel with a similar 50% increase in invasive capacity observed in SW-13/S cells during a 24 h period (*p* < 0.05; Fig. 3b & right panel, Fig. 3c). As can be predicted, opposite to the outcome of enforced SLC12A7 overexpression in SW-13 cells, inhibition of SLC12A7 expression by RNAi gene silencing resulted in a significant reduction of migration and Matrigel-invasion behavior of NCI-H295R cells (Fig. 4a, b, & c, *p* < 0.05). Together these findings suggest that SLC12A7 may play in integral role in ACC cell motility and invasion activity.

### SLC12A7 overexpression promotes cell adhesion and detachment kinetics

**Fig. 5:**
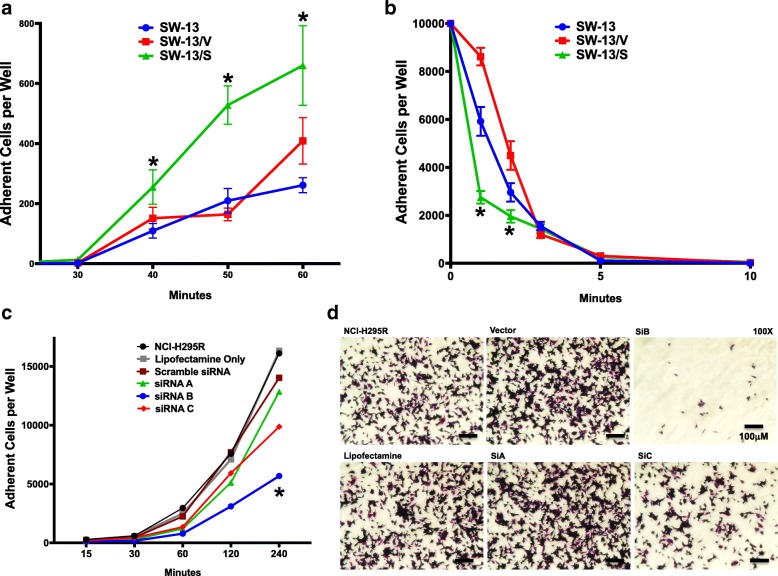
SW-13 cell adhesion and detachment kinetics analysis. **a** Ten thousand cells per well of SW-13, SW-13/V, and SW-13/S were incubated in complete medium (CM) at various time points to allow for cell adherence. After designated time point, cells were washed and attached cells were counted. SW-13/S cells adhered more readily (*, *p* < 0.05, two-way ANOVA). *Horizontal bar,* mean; *Error bars*, standard error of mean. Adhesion assay was performed in 2 independent experiments using 4 wells per each cell type analyzed. **b** Ten thousand cells per well of SW-13, SW-13/V, and SW-13/S were incubated in CM overnight. The following day cells were detached at designated time points with ethylenediaminetetraacetic acid tetrasodium salt dehydrate (Sigma-Aldrich). The cells that remained attached were counted. SW-13/S cells detached more readily (*, *p* < 0.05, two-way ANOVA). *Horizontal bar,* mean; *Error bars*, standard error of mean. Detachment assay was performed in 2 independent experiments using 4 wells per each cell type analyzed. **c** Two hundred fifty thousands cells per well of NCI-H295R undergoing *SLC12A7* RNAi gene silencing (siRNA A, B, C) were incubated in CM for various time points to allow for cell adherence. Parental cells, cells mocked transfected, and cells transfected with scrambled siRNA were used as controls. After each time point, cells were washed and counted. NCI-H295 cells undergoing RNAi gene silencing adhered less readily (*, p < 0.05, two-way ANOVA). Adhesion assay was performed in 2 independent experiments using 3 wells per each cell type analyzed. **d** Photomicrographs of NCI-H295R cells remaining attached after 2 h, fixed, stained with 0.5% crystal violet, and observed under 200X magnification

The malignant behavior of ACC and other cancers is, in part, influenced by the ability of tumor cells to adhere to surrounding surfaces, which in turn could modulate tumor cell survival, migration, and invasion. In the ovarian cancer cell line OVCAR-3, SLC12A7 co-localizes with EZR at the cell surface promoting cell motility and potentially modulating extra-cellular interactions [19]. We determined whether SLC12A7 overexpression modulated extracellular interactions of the cells with their surrounding substratum by testing their comparative adhesion and detachment efficiencies. As shown in Fig. 5a, SW-13/S cells adhered to the substratum at a significantly faster rate compared to SW-13 and SW-13/V cells (*p* < 0.05), indicating that SLC12A7 may promote faster ACC cell adhesion. SLC12A7 also promoted faster detachment of SW-13/S cells as well (*p* < 0.05, Fig. 5b), indicative of a highly dynamic adhesion-detachment interactions of SLC12A7-expressing cells to the surrounding substratum. Similarly, RNAi gene silencing of *SLC12A7* in SLC12A7-expressing NCI-H295R cells significantly perturbed their adhesion capability in a predictably opposite fashion (*p* < 0.05, Fig. 5c). Notably, SLC12A7 gene silencing resulted in a dose-dependent decrease in cell attachment strength, with the NCI-H295R cells affected by the highest knock down of *SLC12A7* (siRNA B) also demonstrating the greatest perturbation of attachment strength (*p* < 0.05, Fig. 5c & d).

### SLC12A7 overexpression leads to increased filopodia formation and co-localization of SLC12A7 with Ezrin

**Fig. 6:**
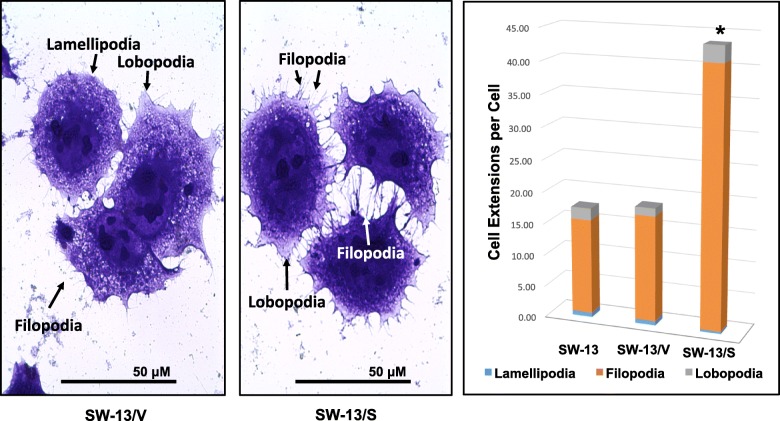
Quantification of cell membrane extensions. Via microscopy, the average number of membrane projections per cell was counted from random photomicrographs (400X). Analysis showed increased filopodia formation (right panel) in SW-13/S cells (middle panel) compared to SW-13 and SW-13/V (left panel) cells (*, *p* < 0.05, two-way ANOVA). *Horizontal bar,* mean; *Error bars*, standard error of mean. Microscopic analysis was performed in duplicate

**Fig. 7:**
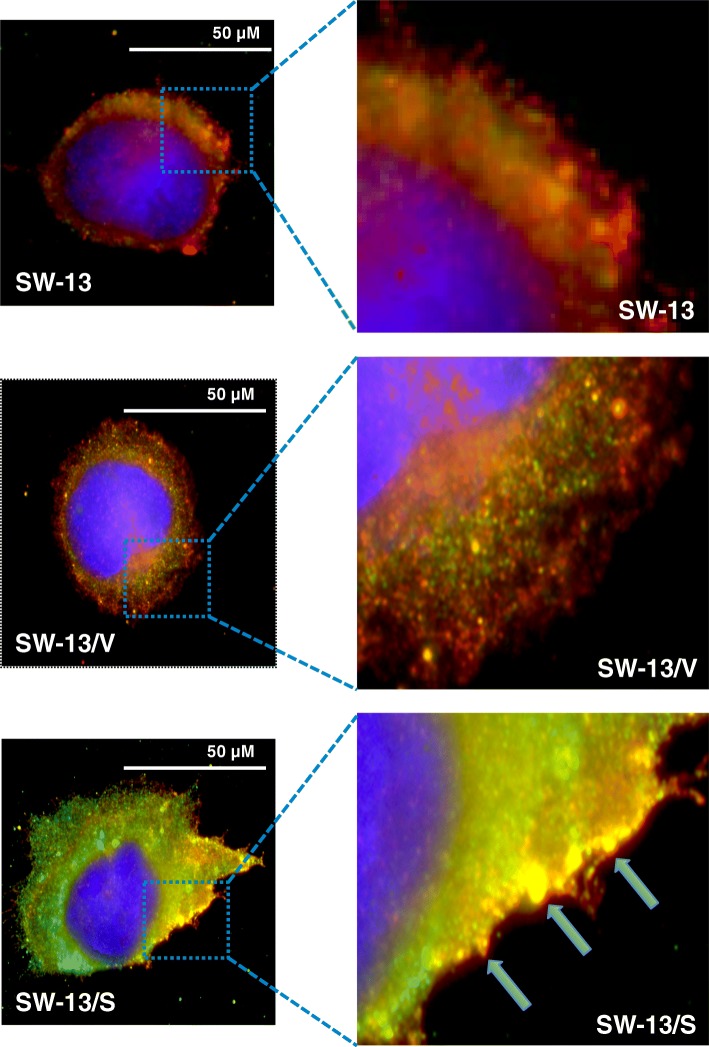
Immunofluorescence analysis of SLC12A7 and Ezrin. Cells were grown on glass cover slips in complete medium and after 24 h cells were fixed in cold acetone-methanol (1:1) for 10 min followed by immunofluorescence detection using anti-SLC12A7 and anti-EZR primary antibodies. Cell nuclei fluoresce blue with DAPI, SLC12A7 fluoresces green, and EZRIN fluoresces red. Filled arrows indicate representative areas of co-localization of SLC12A7 with EZR, which fluoresces yellow. SW-13/S cells have heightened expression of SLC12A7 with co-localization with EZR at the cell membrane, especially at the leading edges of the cell when compared to controls (SW-13 and SW-13/V). Immunofluorescence analysis was performed in duplicate

Under high power (400X) light microscopy, SW-13/S cells appeared to have drastically altered cell membrane extension repertoire (Fig. 6). Assessment of the cell membrane projections associated with cell motility, specifically lamellipodia, lobopodia, and filopodia, showed a distinct difference in their organization in SW-13/S cells compared to SW-13/V cells (left panel, Fig. 6) and parental SW-13 cells (not shown). Microscopic quantification revealed increased filopodia formation, partly at the expense of reduced lamellipodia extensions, in SW-13/S cells compared to parental SW-13 and SW-13/V cells (*p* < 0.05, right panel, Fig. 6). As reported earlier [19], SLC12A7 in SW-13/S cells was seen to have mobilized to the leading edge of the cell’s surface to co-localize with Ezrin, as revealed by immunofluorescence analysis (Fig. 7).

### Transcription factor analysis suggests potential roles for multiple signally pathways in mediating the invasive behavior promoted by SLC12A7 overexpression

**Fig. 8:**
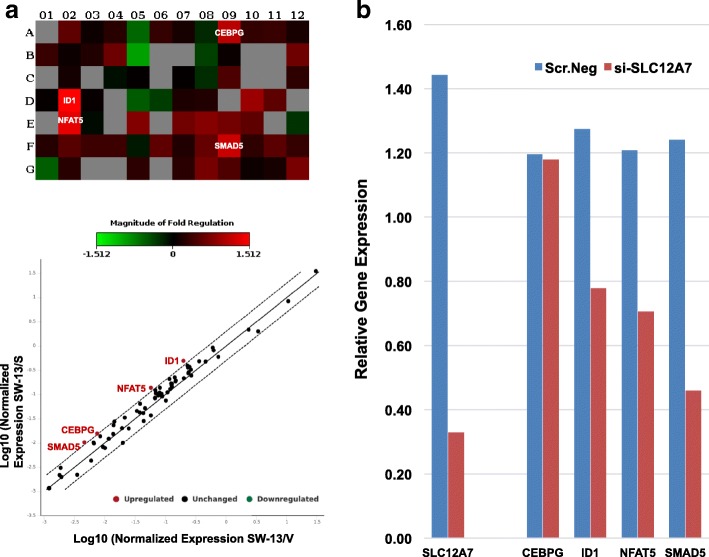
Transcription factor expression analysis. **a** To assess possible altered transcriptional activation downstream to SLC12A7 enforced expression, transcription factor expression analysis was performed using the RT^2^ Profile PCR Human Transcription Factors array. The four transcription factors that appeared to be significantly (*p* = 0.05) overexpressed is noted in the heatmap (above) and the Log10 expression is shown in the scatter plot (below). Note, none of the targeted transcription factors showed a significant down-regulation in SW-13/S cells. Transcription factor expression analysis was performed in duplicate. **b** To confirm the significance of altered transcription factor signaling identified in SW-13/S cells, altered transcription factor expression was measured using quantitative PCR techniques (refer above) in NCI-H295R cells undergoing siRNA knock down. Expressions levels of ID1, NFAT5, and SMAD5 were suppressed, while expression of CEBPG was not significantly altered. Transcription factor expression analysis was performed in duplicate

To assess possible altered transcription factor activation downstream to SLC12A7 enforced expression, comparative transcription factor expression analysis was performed using the targeted RT^2^ Profiler PCR Human Transcription Factors array in SW-13/S and SW-13/V cells. Parental SW-13 cells were used for normalized comparison. Of the targeted 84 transcription factors screened, four transcription factors namely, CEBPG (CCAAT/Enhancer Binding Protein Gamma), ID1 (Inhibitor of DNA binding 1, HLH protein), NFAT5 (Nuclear Factor of Activated T-Cells 5), and SMAD5 (SMAD Family Member 5) were found to be significantly upregulated (> 2 fold) in SW-13/S cells (Fig. 8a). The increased expression of CEBPG, ID1, NFAT5, and SMAD5 transcription factors in SW-13/S cells also suggest their potential role(s) in dysregulating multiple signaling pathways including the Hippo, osmotic stress, and the bone morphogenetic protein (BMP) signaling pathways, directly or indirectly [23–26]. Perturbations in these pathways downstream to SLC12A7 overexpression can potentially alter the adhesion kinetics and invasive properties of ACC cells, as observed in this study.

To confirm the significance of altered transcription factor signaling identified in SW-13/S cells, expression of candidate transcription factor identified to be overexpressed in SW-13/S cells was measured in NCI-H295R cells undergoing siRNA knock down using quantitative RT-PCR (refer above). In line with the previous results, we saw that expressions levels of ID1, NFAT5, and SMAD5 were suppressed (Fig. 8b). Expression level of CEBPG was not significantly altered (Fig. 8b). These findings further show that these transcription factors may indeed play a significant role in SLC12A7 mediated malignant behavior promotion.

## Discussion

Previous studies have demonstrated widespread expression and significant physiologic functions for SlC12A7 in nervous, gastric, and renal tissues [27–30]. However, a physiological role for SLC12A7 in the adrenal cortex has not been addressed. We recently demonstrated moderate expression of SLC12A7 in normal adrenal gland tissue with amplified expression levels observed in ACC tumor samples, suggesting a potential role for SLC12A7 overexpression in ACC tumorigenesis or malignant progression [13]. Similar to its suggested roles in other tissues, basal expression of SLC12A7 may have a physiological role in the organization and function of the adrenal cortex, which when dysregulated may perturb the osmotic status of the cell, and potentially contribute to the accentuated invasive, and consequently more aggressive tumor phenotype seen here.

Previous studies also have shown a clear role for SLC12A7 in promoting in vitro tumor cell invasion. In a study by Hsu et al., RNAi gene silencing of SLC12A7 inhibited breast cancer cell invasion, without any effect on cell viability or proliferation. In the same study, the authors also demonstrated that SLC12A7 activity, in part, promoted in vivo tumor growth in SCID mice [18]. In the present study, we show a similar role for SLC12A7 in promoting cancer cell migration and invasion, phenotypic hallmarks of aggressive cancers, in two well-characterized ACC cell lines, without any significant impact on cell growth or clonogenic potential. The invasion-promoting role of SLC12A7 was confirmed by complementary approaches of enforcing SLC12A7 in cells with undetectable expression (SW-13) and silencing the gene expression in cells with robust expression of SLC12A7 (NCI-H295R).

Moreover, SLC12A7-silencing in NCI-H295R cells showed a dose-response effect on cell adhesion. Transfection with siRNA B, which showed the greatest inhibitory effect on gene expression levels, also had the largest impact on cell attachment compared to other less potent siRNAs. Although highly speculative, this observation suggests that targeting of SLC12A7 in ACC patients may exhibit similar pharmacokinetic effects.

Due to the trans-membrane organization of SLC12A7, with extracellular interactive domains, we speculated that the motility/invasive-promoting effects of SLC12A7 in ACC cells overexpressing SLC12A7 might be mediated by altered adhesive properties consequent to SLC12A7-regulated cell membrane organization. As predicted, the overall adhesion kinetics appeared to be modified to accelerate the motility behavior of SW-13 cells. In SW-13/S cells, lamellipodia formation, which promotes cell spreading and consequently slow cell movement of cells, was found significantly reduced, while motility promoting filopodia was found significantly increased in number, suggesting a cell membrane-associated modulation of cell movement. Moreover, co-localization of SLC12A7 with EZR, a structural and functional linker for cell motility at the leading edges of fast moving SW-13/S cells, further suggests an active role for SLC12A7 in accentuating ACC cell motility and invasive behavior. Alternatively, increased expression of CEBPG and SMAD5, transcription factors with known roles in cancer cell metastasis via Hippo and BMP pathways respectively, suggests roles for additional signaling events in modulating the invasive behavior of SW-13 cells [23, 26]. It is also interesting to note a potential association between the solute carrier SLC12A7 overexpression and upregulation of NFAT5, a transcription factor modulated by osmotic stress, in potentially playing a role in the invasive behavior of ACC cells [25].

Clarification of the probable aggressiveness of SLC12A7 amplification in a larger clinical study cohort would help further clarify the potential translational aspect of these findings. We previously demonstrated that SLC12A7 amplification was associated with hormonally inactive (potentially more de-differentiated) tumors in a smaller discovery cohort of ACC tumors [13]. However, determination of a likely association of SLC12A7 amplifications with tumor stage, invasiveness, and survival needs to be explored in a larger cohort.

## Conclusions

In conclusion, this study demonstrates that SLC12A7 amplification may promote tumor cell migration and invasion in ACC, at least in part by modulating cell membrane organization and perturbed osmotic signaling. Thus, SLC12A7 could represent a molecular marker of ACC tumor aggressiveness and may serve as a potential therapeutic target.

## Abbreviations

ACC: Adrenocortical carcinoma
BMP: Bone morphogenetic protein
CEBPG: CCAAT/Enhancer binding protein gamma
CM: Complete medium
CNV: Copy number variations
DMEM: Dulbecco’s modified eagle medium
ENSAT: European network for the study of adrenal tumors
EZR: Ezrin
FBS: Fetal bovine serum
ID1: Inhibitor of DNA binding 1, HLH protein
IGF-II: Insulin growth factor II
NFAT5: Nuclear factor of activated T-cells 5
PBS: Phosphate-buffered saline
SLC12A7: Solute carrier family 12 member 7
SMAD5: Family member 5
WES: Whole-exome sequencing
